# Comparison between Enzyme Immunoassays Performed on Samples of Dried Blood and Serum for Toxoplasmosis Prenatal Screening: Population-based Study

**DOI:** 10.1055/s-0041-1730285

**Published:** 2021-06-02

**Authors:** Bárbara Araújo Marques, Ericka Vianna Machado Carellos, Vânia Maria Novato Silva, Fernando Henrique Pereira, Maria Regina Lage Guerra, Jacqueline Araújo Domingos Iturra, José Nélio Januário, Gláucia Manzan Queiroz de Andrade

**Affiliations:** 1 Universidade Federal de Minas Gerais, Belo Horizonte, MG, Brazil; 2 Fundação Ezequiel Dias, Belo Horizonte, MG, Brazil

**Keywords:** prenatal care, prenatal diagnosis, dried blood spot testing, toxoplasmosis, congenital toxoplasmosis, cuidado pré-natal, diagnóstico pré-natal, teste em amostras de sangue seco, toxoplasmose, toxoplasmose congênita

## Abstract

**Objective** Most prenatal screening programs for toxoplasmosis use immunoassays in serum samples of pregnant women. Few studies assess the accuracy of screening tests in dried blood spots, which are of easy collection, storage, and transportation. The goals of the present study are to determine the performance and evaluate the agreement between an immunoassay of dried blood spots and a reference test in the serum of pregnant women from a population-based prenatal screening program for toxoplasmosis in Brazil.

**Methods** A cross-sectional study was performed to compare the immunoassays Imunoscreen Toxoplasmose IgM and Imunoscreen Toxoplasmose IgG (Mbiolog Diagnósticos, Ltda., Contagem, Minas Gerais, Brazil)in dried blood spots with the enzyme-linked fluorescent assay (ELFA, BioMérieux S.A., Lyon, France) reference standard in the serum of pregnant women from Minas Gerais Congenital Toxoplasmosis Control Program.

**Results** The dried blood spot test was able to discriminate positive and negative results of pregnant women when compared with the reference test, with an accuracy of 98.2% for immunoglobulin G (IgG), and of 95.8% for immunoglobulin M (IgM).

**Conclusion** Dried blood samples are easy to collect, store, and transport, and they have a good performance, making this a promising method for prenatal toxoplasmosis screening programs in countries with continental dimensions, limited resources, and a high prevalence of toxoplasmosis, as is the case of Brazil.

## Resumo

**Objetivo** A maioria dos programas de triagem pré-natal para toxoplasmose utiliza imunoensaios em amostras de soro de gestantes. Poucos estudos avaliam a acurácia dos testes de triagem em amostras de sangue seco, que são de fácil coleta, armazenamento e transporte. Este estudo teve como objetivo determinar o desempenho e avaliar a concordância entre um imunoensaio em sangue seco e um teste de referência em soro de gestantes de um programa de rastreamento pré-natal de base populacional para toxoplasmose no Brasil.

**Métodos** Realizou-se um estudo transversal para comparar os imunoensaios Imunoscreen Toxoplasmose IgM e Imunoscreen Toxoplasmose IgG (Mbiolog Diagnósticos, Ltda., Contagem, Minas Gerais, Brazil) em sangue seco com o padrão de referência ensaio fluorescente ligado a enzimas ( *enzyme-linked fluorescent assay* , ELFA, BioMérieux S.A., Lion, França) no soro de gestantes do Programa de Controle de Toxoplasmose Congênita de Minas Gerais.

**Resultados** O exame em sangue seco foi capaz de discriminar os resultados positivos e negativos das gestantes quando comparado ao teste de referência, com acurácia de 98,2% para imunoglobulina G (IgG), e de 95,8% para imunoglobulina M (IgM).

**Conclusão** O sangue seco apresenta bom desempenho e é uma amostra de fácil coleta, armazenamento e transporte, o que o torna um método promissor para programas de triagem pré-natal de toxoplasmose em países com dimensões continentais, recursos limitados, e alta prevalência de toxoplasmose, como é o caso do Brasil.

## Introduction

Surveillance of acute toxoplasmosis infections in pregnant women is performed through periodic serological tests obtained from venipuncture. Dried blood spot testing has been successfully used in neonatal screening programs, and may be a promising alternative for the prenatal diagnosis of toxoplasmosis. Collection of dried blood spots is performed through capillary puncture, and the samples are stored in filter paper cards, which are stable and can be transported at low cost, enabling the performance of tests in patients from distant and economically-disadvantaged regions.

In Brazil, studies 1 2 3 from prenatal screening programs for toxoplasmosis based on dried blood spot testing reported a low prevalence of positive immunoglobulin M (IgM) samples, of 0.4% and 0.7%, leading to questions about the accuracy of this test. A systematic review 4 assessed the serological methods used in prenatal screening programs for toxoplasmosis worldwide and did not find publications with dried blood samples.

The present study was conducted in Minas Gerais (MG), the second most populous state in Brazil, located in the Southeastern region of the country. The Minas Gerais Congenital Toxoplasmosis Control Program (MG-CTCP) was implemented by the state's Department of Health, in partnership with Núcleo de Ações e Pesquisa em Apoio Diagnóstico (NUPAD, in Portuguese) of the School of Medicine at Universidade Federal de Minas Gerais (UFMG) in February 2013. The MG-CTCP included prenatal screening for toxoplasmosis of all pregnant women from 853 municipalities in the state by using dried blood on filter paper, which made universal screening possible in MG. There were ∼ 270,000 pregnant women in MG in 2014. More than 95% of them received prenatal care, and 74% attended 7 or more visits. Approximately 70% of pregnant women in the state are users of the Brazilian Unified Health System (Sistema Único de Saúde, SUS, in Portuguese), and NUPAD tested around 60% of them in 2014.

The primary objective of the present cross-sectional study was to compare the performance of a dried blood test used in the MG-CTCP with that of a commercial serological test considered a reference for the diagnosis of infection. The secondary objective was to determine the prevalence of toxoplasmosis among the pregnant women in the MG-CTCP.

## Methods

A cross-sectional study was performed with pregnant women in the MG-CTCP who were cared for in one of the 853 municipalities in the state of MG. The sample size was calculated as 1,000 pregnant women to find a minimum number of 5 acute infections among them, as it is estimated that 40% of pregnant women in Brazil are susceptible to toxoplasmosis, and a cumulative incidence of seroconversion in pregnancy from 4.8 to 5.7 per 1000 is described. 5 6 Additionally, we planned to include 40 pregnant women with positive IgM results via filter paper, detected by the MG-CTCP in the same period, which is enough to reduce the low-frequency bias expected in the 2 × 2 tables. The Basic Health Units (BHUs) included were randomly selected in each of the 13 macroregions of MG, considering the proportion of pregnant women with an acute infection profile (positive IgM and immunoglobulin G [IgG] anti- *Toxoplasma gondii* ) among all pregnant women in the MG-CTCP in the year before the study, and the average amount of screenings performed per month and per BHU in the same period, according to the NUPAD database. A macroregion with a higher prevalence of probable acute infections had a larger number of patients screened than a region with a lower prevalence. Likewise, a BHU that performed a higher number of screening tests in the base period had a higher probability of being drawn within each macroregion. This method is called sampling with probabilities proportional to size. By rounding off issues in the monthly average number of examinations conducted by the BHUs, the total amount of samples was calculated as 1,038.

Pregnant women who refused to participate in the study, as weell as those who presented unsuitable samples, were excluded. Pregnant women were included consecutively at the time of the first blood collection. Capillary and venous blood samples were collected simultaneously or with a difference of up to 24 hours, and were respectively transported on Whatman (Merck KGaA, Darmstadt, Germany and/or its affiliates. Sigma-Aldrich, Inc.) 903 filter paper at room temperature, and in styrofoam boxes with ice to the NUPAD reference laboratory. Free and informed consent was obtained for all pregnant women who agreed to participate in the study.

The present study was blinded regarding the professionals in charge of conducting the serological tests, but not regarding the main researchers, who needed to adopt clinical procedures of interest to the patient.

Immunoassays were used to determine anti- *T. gondii* specific IgM and IgG antibodies: the Imunoscreen (Mbiolog Diagnósticos, Ltda., Contagem, Minas Gerais, Brazil) in dried blood samples in the NUPAD laboratory, and the enzyme-linked fluorescent assay (ELFA, BioMérieux S.A., Lyon, France) in serum, using the automated VIDAS system in two laboratories outsourced by NUPAD (A and B). The IgG avidity test was performed using the serum of all pregnant women with reagent IgG results: chemiluminescence and ELFA by laboratories A and B respectively.

The Statistical Package for the Social Sciences (SPSS, SPSS, Inc., Chicago, IL, US) software, version 18, was used to set up a database and perform the statistical analyses. The performance of the Imunoscreen test in filter paper was evaluated in comparison with the reference standard (ELFA), and its sensitivity, specificity, positive predictive value (PPV), and negative predictive value (NPV) were calculated. For each of these parameters, additional analyses were performed, excluding the indeterminate results, or considering them as positive or negative. The receiver operating characteristic (ROC) curve was used to describe the performance of dried blood tests to classify pregnant women as infected or not by *T. gondii* .

The Chi-squared test for adherence was used to evaluate whether the distribution of samples received from each macroregion matched the distribution of the expected samples. The prevalence of toxoplasmosis was determined by the proportion of pregnant women with positive IgG anti- *T. gondii* results compared with the total number of pregnant women screened.

The Kappa coefficient was used to evaluate the agreement between the results of the tests in filter paper and serum samples. Since the Kappa depends on the agreement that goes beyond the coincidence of random evaluations, it is possible to find low values of this measurement due to the low prevalence of the event. It is usually associated with low levels of reproducibility, not due to substantial errors related to the test. 7 Therefore, the prevalence was calculated using adjusted Kappa for IgM.

The research project was approved by the Ethics in Research Committee of UFMG (CEP-257.199, 26/04/2013) and received funding from Conselho Nacional de Desenvolvimento Científico e Tecnológico (CNPq, Brazilian National Council for Scientific and Technological Development) (Case 456491/ 2014–7). Activity planning was supported by the institutions involved.

## Results

From July 1, 2014, to December 31, 2014, we received 750 samples of pregnant women participating in the MG-CTCP. Of these, 40 were excluded because they were inappropriate; therefore, 710 pregnant women remained in the study. The Chi-squared test for adherence showed that the distribution of the 710 included samples was statistically similar to the distribution of the expected 1,038 samples concerning their origin by macroregion ( *p* = 0.126). The prevalence of toxoplasmosis among the 710 pregnant women studied was of 45.2%. None had positive IgM test results. In total, 33 pregnant women with positive IgM samples in filter paper, detected in the MG-CTCP in the same screening period, were included in the study, totaling 743 pregnant women. The age of the sample ranged from 12 to 47 years (median: 24 years). The first examination was performed at a median gestational age of 10 weeks, and the median interval in days between the collection of dried blood and serum samples was zero. Considering the ELFA method as a reference test, the relative sensitivity, specificity, PPV, and NPV of the Imunoscreen test for the detection of IgM and IgG antibodies in dried blood were calculated ( Table 1 ).

**Table 1:** Distribution of IgM and IgG results in dried blood and serum samples in 743 pregnant women participating in the comparison between the Imunoscreen test (dried blood) and the ELFA (serum) reference test

Tests			Result by the ELFA B method in serum			
		Results	Positive	Indeterminate	Negative	Total
Result by the Imunoscreen A method in filter paper	IgM	Positive	21	2	10 **	33
		Indeterminate	1	1	1	3
		Negative	15 *	3	689	707
		Total	37	6	700	743
	IgG	Positive	321	0	9 ##	330
		Indeterminate	49	0	2	51
		Negative	12 #	1	346	359
		Total	382	1	357	740

Abbreviations: ELFA, enzyme-linked fluorescent assay; IgG, immuniglobulin G; IgM, immunoglobulin M.

Notes:

* False negative IgM.

** False positive IgM.

# False negative IgG.

## False positive IgG.

A Imunoscreen test. According to the manufacturer, the test presents 100% of sensitivity and 98.1% of specificity for IgM, and 100% and 99.1% respectively for IgG.

B Enzime-linked fluorescent assay (ELFA). According to the manufacturer, the test presents 81.8% to 90.9% of sensitivity for IgM, and 93.8% to 98.4% for IgG, and a specificity of 100.0% for IgM and of > 99.0% for IgG. The IgM results are interpreted as negative if lower than 0.55, indeterminate if between 0.55 and 0.64, and positive if ≥0.65. For IgG, the result is negative if lower than 4.0, intermediate if between 4.0 and 7.9, and positive if ≥ 8.0.

No significant difference in IgM sensitivity of the filter paper test was observed when indeterminate results were excluded (58.3%; 95% confidence interval [95%CI]: 42.2–72.9%) or included among the positive results (58.1%; 95%CI: 43.3–71.6%) ( *p* = 1.000) ( Table 2 ). However, a significant reduction in IgG sensitivity of the Imunoscreen test was observed when indeterminate results were included among the negative results (56.8%; 95%CI: 40.9–71.3%; *p* < 0.001).

**Table 2:** Sensitivity, specificity, positive predictive value (PPV), negative predictive value (NPV) and Kappa concordance coefficient for the Imunoscreen test (dried blood) compared with the ELFA (serum) reference test, considering indeterminate results in the sample analyzed to be positive

Imunoscreen test (dried blood) compared with the ELFA (serum)	IgM	IgG
Sensitivity – % (95%CI)	58.1 (43.3–71.6)	96.6 (94.3–98.0)
Specificity – % (95%CI)	98.4 (97.2–99.1)	96.9 (94.6–98.3)
PPV – % (95%CI)	69.4 (54.4–84.5)	97.1 (95.4–98.8)
NPV – % (95%CI)	97.5 (96.3–98.6)	96.4 (94.4–98.3)
Kappa coefficient of agreement (95%CI)	0.61 (0.55–0.68)	0.93 (0.92–0.95)
Kappa adjusted for IgM prevalence (95%CI)	0.92 (0.91–0.94)	–

Abbreviations: 95%CI, 95% confidence interval; ELFA, enzyme-linked fluorescent assay; IgG, immunoglobulin G; IgM, immunoglobulin M.

The Kappa coefficient of agreement in serum and dried blood was calculated for IgM, IgG, and IgM when adjusted for prevalence ( Table 2 ). When the indeterminate results were included as positive, a good agreement was found between the tests for IgM, and a very good agreement for IgG. When prevalence-adjusted Kappa for IgM was used, a very good agreement between the tests was found, regardless of the inclusion of indeterminate results.

There were 15 false-negatives for IgM in filter paper. In each case, dried blood and serum samples were collected on the same day or up to 24 hours after. In 10 out of 13 cases with information on gestational age, the sample was collected in the first trimester of gestation. All samples tested positive for IgG, except for a dried blood sample with indeterminate IgG. There was low avidity of IgG only in 1 case. In addition, 14 samples presented serum IgM values extremely close to the cut-off of the test. Toxoplasma infection was excluded in all the children of these mothers after the follow-up.

There were 10 false-positive results for IgM and 9 false-positives for IgG. There were also 12 false-negative results for IgG in filter paper, all of which had low values of IgG in serum, high IgG avidity, and negative IgM. Serology was repeated during the routine prenatal follow-up for all of these 12 subjects.

The ROC curves showed that the Imunoscreen test could discriminate positive and negative pregnant women when compared with the reference test, with the area under the curve equal to 98.2% for IgG and to 95.8% for IgM.

The IgM screening was performed as part of the MG-CTCP in newborns of susceptible mothers or of mothers with suspected acute infection during pregnancy. The results of neonatal screening were available for 316 of the 419 newborns of 412 pregnant women with positive IgM in pregnancy; all of them were negative.

## Discussion

This population-based study compared serologies performed on two types of blood samples (dried blood on filter paper and serum) in a large cohort of pregnant women participating in a longitudinal toxoplasmosis screening program.

The prevalence of toxoplasmosis found, 45.2%, was compatible with the overall results of the MG-CTCP, but lower than previous findings in the city of Belo Horizonte and in Brazil. 8 9

None of the 710 pregnant women initially included in the study had positive IgM results. In Brazil, some studies report a rate of up to 8% of acute infection in pregnancy, but the IgM antibody is generally observed in ∼ 0.5% to 2.0%. 10 11 Boa-Sorte et al. 12 used the Imunoscreen test and reported 1.88% (95%CI: 0.6–2.71) of positive IgM results among pregnant women, which suggests that the sample of the present study may have been insufficient to identify acute cases. As the parameters of sensitivity, specificity, and ROC curve are independent of the prevalence of the studied event, it was possible to add IgM reagent samples to the total of samples originally calculated and to carry out an analysis of IgM results. 13

The test under study was compared with a reference test which, although presents high sensitivity and specificity, may not represent all of the true positive and negative results of the individuals. The unavailability of a “gold standard” test for the detection of IgM antibodies to toxoplasma makes it difficult to evaluate new tests, and may perpetuate inherent errors in the reference test. Several investigators have already shown that the accuracy of the tests differ markedly, depending on the use of selected or routine sera. 14 Therefore, positive IgM test results should be confirmed by additional tests (such as the IgM immunosorbent agglutination assay [ISAGA] and differential agglutination, for example) in laboratories experienced in the diagnosis of toxoplasmosis, or by demonstration of a significant increase in antibody titers in serial serum samples with intervals of at least 3 weeks that run parallel to prevent misinterpretation. 6 15

There were 12 false-negative IgG results in filter paper. When analyzed, the serum results were compatible with those of long-term acquired infection, that is, low IgG, non-reactive IgM, and high avidity of IgG. As these pregnant women were considered susceptible according to the filter paper results, the serology was repeated during the routine prenatal follow-up, which allowed for diagnostic opportunities. In total, 9 cases of false-positive IgG results in filter paper were observed, but a serological follow-up was not performed, since those pregnant women were considered infected before pregnancy. Although they represented only 1.2% of the total cases, this occurrence should be minimized as much as possible.

A total of 10 pregnant women with false-positive IgM results in filter paper had mandatory confirmation of serology in the serum samples, and the results could be correctly reclassified.

When the indeterminate results were included as positive ( Table 2 ), a good agreement was found between the Imunoscreen and the reference tests for IgM, and a very good agreement for IgG, a plausible option at the MG-CTCP workflow, which repeats all the exams with indeterminate results. When the prevalence-adjusted Kappa for IgM was used, as this antibody has low prevalence (1% to 2%) in the population, as observed in the present study, a very good agreement between the tests was found, regardless of the inclusion of indeterminate results. 15 However, some authors argue that the value of Kappa obtained with adjustment according to prevalence may not reflect reality, and may suggest that corrections must be made regarding sample size. 16 On the other hand, lower Kappa values for IgG and lower IgG sensitivity in filter paper were observed when indeterminate results were included among the negatives, with significant difference. Likewise, most of the indeterminate IgG antibodies detected corresponded to positive values in low titers, from past infections, or even from early infections in a few cases. This finding suggests the need for adequacy in the cut-off initially used; however, this did not represent a problem for the MG-CTCP, since indeterminate results were always confirmed with a new serum sample.

## Conclusion

Considering the high prevalence of toxoplasmosis in Brazil, the risk of acute infection in pregnant women, and the severity of congenital toxoplasmosis, screening for infection enables adequate prevention and may be cost-effective. The good performance of dried blood samples makes this a promising method for countries with continental dimensions, limited resources, and high prevalence of toxoplasmosis, such as Brazil.

## References

[1] Figueiró-Filho E A Lopes A HA Seneforte F RA de Souza V G Júnior Botelho C A Figueiredo M S Duarte G [Acute toxoplasmosis: study of the frequency, vertical transmission rate and the relationship between maternal-fetal diagnostic tests during pregnancy in a Central-Western state of Brazil] Rev Bras Ginecol Obstet 2005 27 08 442 449 10.1590/S0100-72032005000800002

[2] Sartori A L Minamisava R Avelino M M Martins C A [Prenatal screening for toxoplasmosis and factors associated with seropositivity of pregnant women in Goiânia, Goiás] Rev Bras Ginecol Obstet 2011 33 02 93 98 10.1590/S0100-72032011000200007 21779652

[3] Amaral E [A population screening program for toxoplasmosis?] Rev Bras Ginecol Obstet 2005 27 08 439 441 10.1590/S0100-72032005000800001

[4] Marques B A Andrade G MQ Neves S PF Pereira F H Talim M CT [Systematic review of serological methods used in prenatal screening for toxoplasmosis in pregnant women] Rev Med Minas Gerais. 2015 25 06 S68 S81 10.5935/2238-3182.2015009

[5] Couto J CF Avelino M M Ferreira Q TM [Toxoplasmosis: toxoplasmosis and pregnancy] Rio de Janeiro Guanabara Koogan 2006 471 492

[6] Peyron F Wallon M Kieffer F Garweg J Toxoplasmosis 8th ed. Philadelphia Elsevier Saunders 2015 949 1042

[7] Brito C Portela M C de Vasconcellos M T [Clinical and demographic data concordance comparing authorizations for high-complexity oncological procedures and patient records of women treated under the Unified National Health System in Rio de Janeiro, Brazil] Cad Saude Publica 2005 21 06 1829 1835 10.1590/s0102-311x2005000600032 16410869

[8] Carellos E VM Andrade G MQ Aguiar R ALP [Evaluation of prenatal screening for toxoplasmosis in Belo Horizonte, Minas Gerais State, Brazil: a cross-sectional study of postpartum women in two maternity hospitals] Cad Saude Publica 2008 24 02 391 401 10.1590/s0102-311x2008000200018 18278286

[9] Dubey J P Lago E G Gennari S M Su C Jones J L Toxoplasmosis in humans and animals in Brazil: high prevalence, high burden of disease, and epidemiology Parasitology 2012 139 11 1375 1424 10.1017/S0031182012000765 22776427

[10] Avelino M M Parada J CB Castro A M Alves M FC Campos Júnior D Toxoplasma gondii primary infection in pregnant women in Goiânia: a seroconversion study Rev Ciênc Méd Biol 2009 8 03 325 333

[11] Varella I S Canti I CT Santos B R Coppini A Z Argondizzo L C Tonin C Wagner M B Prevalence of acute toxoplasmosis infection among 41,112 pregnant women and the mother-to-child transmission rate in a public hospital in South Brazil Mem Inst Oswaldo Cruz 2009 104 02 383 388 10.1590/S0074-02762009000200037 19430669

[12] Boa-Sorte N Purificação A Amorim T Assunção L Reis A Galvão-Castro B Dried blood spot testing for the antenatal screening of HTLV, HIV, syphilis, toxoplasmosis and hepatitis B and C: prevalence, accuracy and operational aspects Braz J Infect Dis 2014 18 06 618 624 10.1016/j.bjid.2014.05.009 25022566 PMC9425210

[13] Martinez E Z Louzada Neto F Pereira B B [Analysis of diagnostic tests using ROC curves] Cad Saude Colet 2003 11 01 7 31

[14] Liesenfeld O Press C Montoya J G Gill R Isaac-Renton J L Hedman K Remington J S False-positive results in immunoglobulin M (IgM) toxoplasma antibody tests and importance of confirmatory testing: the Platelia Toxo IgM test J Clin Microbiol 1997 35 01 174 178 10.1128/JCM.35.1.174-178.1997 8968902 PMC229533

[15] Flori P Chene G Varlet M N Sung R T [Toxoplasma gondii serology in pregnant woman: characteristics and pitfalls] Ann Biol Clin (Paris) 2009 67 02 125 133 10.1684/abc.2009.0308 19297286

[16] Hoehler F K Bias and prevalence effects on kappa viewed in terms of sensitivity and specificity J Clin Epidemiol 2000 53 05 499 503 10.1016/s0895-4356(99)00174-2 10812322

